# Multifactorial White Matter Damage in the Acute Phase and Pre-Existing Conditions May Drive Cognitive Dysfunction after SARS-CoV-2 Infection: Neuropathology-Based Evidence

**DOI:** 10.3390/v15040908

**Published:** 2023-03-31

**Authors:** Ellen Gelpi, Sigrid Klotz, Miriam Beyerle, Sven Wischnewski, Verena Harter, Harald Kirschner, Katharina Stolz, Christoph Reisinger, Elisabeth Lindeck-Pozza, Alexander Zoufaly, Marlene Leoni, Gregor Gorkiewicz, Martin Zacharias, Christine Haberler, Johannes Hainfellner, Adelheid Woehrer, Simon Hametner, Thomas Roetzer, Till Voigtländer, Gerda Ricken, Verena Endmayr, Carmen Haider, Judith Ludwig, Andrea Polt, Gloria Wilk, Susanne Schmid, Irene Erben, Anita Nguyen, Susanna Lang, Ingrid Simonitsch-Klupp, Christoph Kornauth, Maja Nackenhorst, Johannes Kläger, Renate Kain, Andreas Chott, Richard Wasicky, Robert Krause, Günter Weiss, Judith Löffler-Rag, Thomas Berger, Patrizia Moser, Afshin Soleiman, Martin Asslaber, Roland Sedivy, Nikolaus Klupp, Martin Klimpfinger, Daniele Risser, Herbert Budka, Lucas Schirmer, Anne-Katrin Pröbstel, Romana Höftberger

**Affiliations:** 1 Division of Neuropathology and Neurochemistry, Department of Neurology, Medical University of Vienna, 1090 Vienna, Austria; sigrid.klotz@meduniwien.ac.at (S.K.); christine.haberler@meduniwien.ac.at (C.H.); johannes.hainfellner@meduniwien.ac.at (J.H.); adelheid.woehrer@meduniwien.ac.at (A.W.); simon.hametner@meduniwien.ac.at (S.H.); thomas.roetzer@meduniwien.ac.at (T.R.); till.voigtlaender@meduniwien.ac.at (T.V.); verena.endmayr@meduniwien.ac.at (V.E.); carmen.haider@meduniwien.ac.at (C.H.); judith.ludwig@meduniwien.ac.at (J.L.); andrea.polt@meduniwien.ac.at (A.P.); gloria.wilk@meduniwien.ac.at (G.W.); susanne.schmid@meduniwien.ac.at (S.S.); irene.erben@meduniwien.ac.at (I.E.); anita.nguyen@meduniwien.ac.at (A.N.); thomas.berger@meduniwien.ac.at (T.B.); herbert.budka@meduniwien.ac.at (H.B.); 2 Comprehensive Center for Clinical Neurosciences & Mental Health, Medical University of Vienna, 1090 Vienna, Austria; 3 Departments of Neurology, Biomedicine and Clinical Research, University Hospital and University of Basel, 4031 Basel, Switzerland; miriam.beyerle@unibas.ch (M.B.); anne-katrin.proebstel@usb.ch (A.-K.P.); 4 Research Center for Clinical Neuroimmunology and Neuroscience Basel (RC2NB), Department of Clinical Research, University Hospital and University of Basel, 4031 Basel, Switzerland; lucas.schirmer@medma.uni-heidelberg.de; 5 Department of Neurology, Medical Faculty Mannheim, Heidelberg University, 68167 Mannheim, Germany; s.wischnewski@stud.uni-heidelberg.de; 6 Mannheim Center for Translational Neuroscience and Institute for Innate Immunoscience, Medical Faculty Mannheim, Heidelberg University, 68167 Mannheim, Germany; 7 Department of Pathology, Klinik Favoriten, 1100 Vienna, Austriaharald.kirschner@wienkav.at (H.K.); roland.sedivy@gesundheitsverbund.at (R.S.); martin.klimpfinger@meduniwien.ac.at (M.K.); 8 Department of Forensic Medicine, Medical University of Vienna, 1090 Vienna, Austria; katharina.stolz@meduniwien.ac.at (K.S.); christoph.reisinger@meduniwien.ac.at (C.R.); nikolaus.klupp@meduniwien.ac.at (N.K.); daniele.risser@meduniwien.ac.at (D.R.); 9 Department of Neurology, Klinik Favoriten, 1100 Vienna, Austria; elisabeth.lindeck-pozza@gesundheitsverbund.at; 10 Intensive Care Unit, Klinik Favoriten, 1100 Vienna, Austria; alexander.zoufaly@gesundheitsverbund.at; 11 Faculty of Medicine, Sigmund Freud University, 1020 Vienna, Austria; 12 D&F Institute of Pathology, Neuropathology, Medical University Graz, 8036 Graz, Austria; marlene.leoni@medunigraz.at (M.L.); gregor.gorkiewicz@medunigraz.at (G.G.); matin.zacharias@medunigraz.at (M.Z.); martin.asslaber@medunigraz.at (M.A.); 13 Department of Pathology, Medical University of Vienna, 1090 Vienna, Austria; susanna.lang@meduniwien.ac.at (S.L.); ingrid.simonitsch-klupp@meduniwien.ac.at (I.S.-K.); christoph.kornauth@meduniwien.ac.at (C.K.); maja.nackenhorst@meduniwien.ac.at (M.N.); renate.kain@meduniwien.ac.at (R.K.); 14 Münchner Leukämielabor, 81377 Munich, Germany; 15 Institute of Pathology, Klinik Ottakring, 1160 Vienna, Austria; andreas.chott@gesundheitsverbund.at (A.C.); richard.wasicky@gesundheitsverbund.at (R.W.); 16 Division of Infectious Diseases, Department of Internal Medicine, Medical University of Graz, 8036 Graz, Austria; robert.krause@medunigraz.at; 17 Department of Internal Medicine and Pulmonology, Medical University of Innsbruck, 6020 Innsbruck, Austria; guenter.weiss@i-med.ac.at (G.W.); judith.loeffler@i-med.ac.at (J.L.-R.); 18 Department of Neurology, Medical University of Vienna, 1090 Vienna, Austria; 19 Department of Neuropathology, Tirol Kliniken GmbH, 6020 Innsbruck, Austria; patrizia.moser@meduniwien.ac.at (P.M.); afschin.soleiman@innpath.at (A.S.); 20 Interdisciplinary Center for Neurosciences, Heidelberg University, 69120 Heidelberg, Germany

**Keywords:** COVID-19, neuropathology, white matter, leukoencephalopathy, SARS-CoV-2

## Abstract

Background: There is an urgent need to better understand the mechanisms underlying acute and long-term neurological symptoms after COVID-19. Neuropathological studies can contribute to a better understanding of some of these mechanisms. Methods: We conducted a detailed postmortem neuropathological analysis of 32 patients who died due to COVID-19 during 2020 and 2021 in Austria. Results: All cases showed diffuse white matter damage with a diffuse microglial activation of a variable severity, including one case of hemorrhagic leukoencephalopathy. Some cases revealed mild inflammatory changes, including olfactory neuritis (25%), nodular brainstem encephalitis (31%), and cranial nerve neuritis (6%), which were similar to those observed in non-COVID-19 severely ill patients. One previously immunosuppressed patient developed acute herpes simplex encephalitis. Acute vascular pathologies (acute infarcts 22%, vascular thrombosis 12%, diffuse hypoxic–ischemic brain damage 40%) and pre-existing small vessel diseases (34%) were frequent findings. Moreover, silent neurodegenerative pathologies in elderly persons were common (AD neuropathologic changes 32%, age-related neuronal and glial tau pathologies 22%, Lewy bodies 9%, argyrophilic grain disease 12.5%, TDP43 pathology 6%). Conclusions: Our results support some previous neuropathological findings of apparently multifactorial and most likely indirect brain damage in the context of SARS-CoV-2 infection rather than virus-specific damage, and they are in line with the recent experimental data on SARS-CoV-2-related diffuse white matter damage, microglial activation, and cytokine release.

## 1. Introduction

The COVID-19 pandemic is placing a severe burden on socioeconomic, physical, and mental health. The mid- and long-term consequences are expected to be challenging for the coming years. Neurological symptoms and complications have been a focus of attention for both acute-phase symptoms (e.g., headache, hyposmia, cerebrovascular disease with ischemic and hemorrhagic lesions, symptoms associated with encephalitis mimics, seizures, and cranial and peripheral nerve damage, including Guillain–Barré syndrome) and the sequel of a SARS-CoV-2 infection, termed “Long- or Post-COVID-19“ syndrome that includes various symptoms such as fatigue, memory, and attention problems (“brain fog”), potentially progressive cognitive decline [1,2,3] and autonomic dysfunction.

Neuropathological investigations have contributed to a better understanding of the underlying mechanisms and, thus, the symptoms and complications which occur during the acute phase of the disease [1,2,3,4,5,6,7,8,9,10,11,12,13,14,15,16,17].

We therefore performed a postmortem neuropathological assessment of a series of Austrian patients, who died of/with COVID-19 infection, with the aim of identifying underlying brain, spinal, and neuromuscular pathologies, and to discuss potential substrate(s) of mid- and long-term cognitive alterations as have been reported for post-COVID-19 patients [18,19,20,21].

## 2. Materials and Methods

The study cohort included 32 patients (19 male, 13 female), aged 21 to 97 years at death (mean 65.8, median 66.5), who died during the pandemic in 2020 and 2021. Inclusion criteria were having had a positive PCR for SARS-CoV-2 before death and an autopsy including brain extraction. No exclusion criteria were applied. Details on the premorbid conditions of the patients and cause of death are summarized in Table 1.

Brains were obtained at autopsy at several Austrian Pathology Departments (Klinik Favoriten and Klinik Ottakring in Vienna, University Hospital Graz, University Hospital Innsbruck), at the Department for Forensic Medicine in Vienna, and by Dr. Denk (unaffiliated forensic medicine expert). Ethical approval for the use of human postmortem brain tissue for research purposes was obtained from the Medical University of Vienna (EK1454/2018). The study was performed in accordance with the World Medical Association Declaration of Helsinki.

For the neuropathological examination, we assessed formalin-fixed and paraffin-embedded brain tissues from multiple brain areas, including olfactory bulb and olfactory tract, frontal, temporal, parietal, and occipital cortex, anterior and posterior basal ganglia, amygdala, anterior, and posterior hippocampus, posterior hypothalamus, including the corpora mamillaria, thalamus, and supra- and infratentorial white matter, midbrain, pons, medulla oblongata, cervical spinal cord, cerebellar vermis, and hemispheres with white matter and dentate nucleus. In a subgroup of patients, skeletal muscle and the peripheral nerve were also obtained. As muscles were obtained at autopsy from different departments, only formalin-fixed muscle tissue was available for the study. Muscle type differentiation was performed by immunohistochemistry, with antibodies directed against slow (type I) and fast (type II) myosin, acknowledging that some fibers co-express both.

Five-micrometer-thick sections were stained with hematoxylin-eosin, luxol fast blue (LFB), and, in selected cases and brain areas, Elastica van Gieson combined stain. Immunohistochemistry was performed in selected brain areas using a panel of primary antibodies (see Supplementary Table S1) on a DAKO autostainer (Dako, Glostrup, Denmark). For the visualization of an antigen–antibody reaction, we applied the Dako Envision Kit and diaminobenzidine as chromogen.

A total of 32 cases had a PCR-confirmed SARS-CoV-2 infection either in vivo (30 cases) or postmortem (2 cases), and two thirds had a clinical diagnosis of COVID-19 pneumonia that required intensive care unit treatment (Table 1); no cases with “Post-COVID-19” were studied. We also assessed three additional brains of patients who died in 2009/2010 due to influenza virus (H1N1) infection and compared the distribution of T- and B-cells, microglial reactivity, and additional neuropathological changes.

The assessment of the inflammatory reaction (anti-CD8, anti-CD20, and anti-HLA-DR) was graded in each brain area separately for perivascular spaces and within the parenchyma in a semiquantitative manner, as follows: 0 = absent (no cellular infiltrates); 1 = mild (<5 cells in 1 mm^2^); 2 = moderate (5–10 cells/mm^2^); 3 = abundant (>10 cells/mm^2^). This scale refers to the density of the labeled cells for CD8 and CD20, and for HLA-DR, in addition to the density, we assessed the change in the morphology: in the perivascular space: 1 = mild (<50 cells in 1 mm^2^, rod to delicate ramified morphology); 2 = moderate (50–100 cells/mm^2^, moderate enlargement with coarse branching); and 3 = abundant (>50–100 cells/mm^2^, broad amoeboid morphology). In the parenchyma: 1 = mild (<200 cells/mm^2^, delicate ramified morphology); 2 = moderate (200–500 cells/mm^2^, moderate enlargement with broad branching); and 3 = abundant (>200–500 cells/mm^2^, broad amoeboid morphology). A comparison between patients who died from COVID-19 and patients who died from other conditions who additionally tested positive for SARS-CoV-2 was performed.

The evaluation of white matter changes was based on HE, Luxol Fast Blue, and HLA-DR stains and it was semi-quantitatively assessed in the regions of interest (see below) as: 0 = absent (no obvious vacuoles; no white matter pallor and homogeneous intense blue myelin staining in LFB), + = mild (<5 vacuoles/mm^2^, patchy and smooth discoloration in LFB stain), ++ = moderate (5–10 vacuoles/mm^2^, partly confluent areas of white matter discoloration in LFB stain), and +++ = severe (>10 vacuoles/mm^2^, diffuse confluent reduction in blue intensity in LFB). On HE and LFB, we assessed the degree of pallor and vacuolation, on HLA-DR the density and morphology of microglia in the white matter of the main lobes, frontal, temporal, parietal, and occipital, corpus callosum, and cerebellum, and integrated them in an overall score of “white matter damage”. CD8, CD20, and HLA-DR were assessed for their perivascular and parenchymal compartment separately. As these patients were in the acute phase of disease and most of them were in the intensive care unit, a detailed correlation with neurological symptoms was not possible.

In one patient with concomitant herpes simplex virus type 1 infection, we performed an ultrastructural analysis of the viral particles with a Zeiss electron microscope. For this, a small fragment of formalin-fixed temporal cortex was embedded in Epon resin, cut ultrathin, and contrasted with uranyl acetate.

## 3. Results

**Figure 1:**
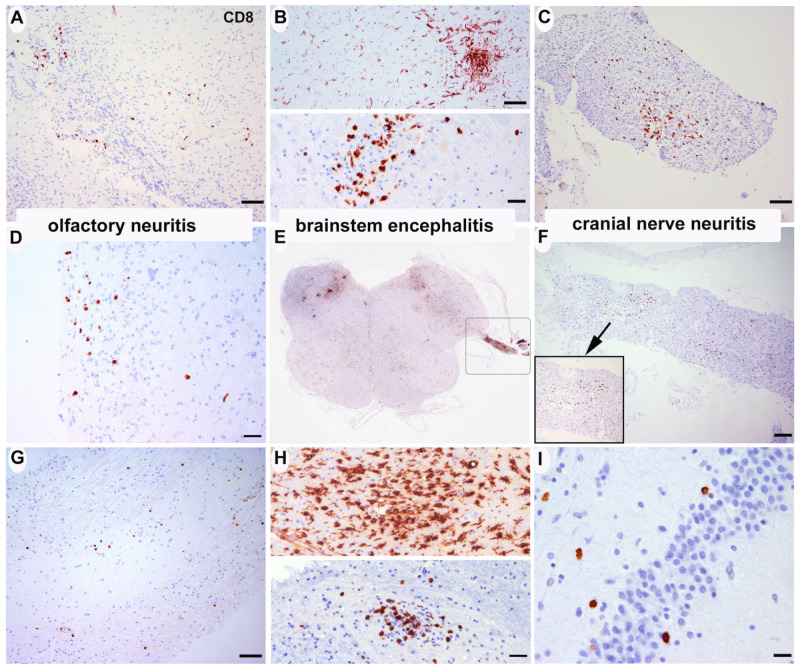
Inflammatory changes. (**A**,**D**,**G**) Focal cellular infiltrates of CD8+ T-lymphocytes in the olfactory bulb/tract (**A**), case 2; (**D**), case 5; (**G**), case 22) (anti-CD8 immunohistochemistry). (**B**,**E**,**H**) Focal nodular accumulation of CD8+ T-lymphocytes and HLA-DR+ microglia in the medulla oblongata (**B**), upper panel anti-HLA-DR, lower panel anti-CD8, case 3; (**E**), anti-HLA-DR, case 5; (**H**), upper panel anti-HLA-DR, lower panel anti-CD8, case 4). (**C**,**F**) Focal cellular infiltrates of CD8+ T-lymphocytes along emerging cranial nerves (vagus) (**C**), case 2; (**F**), case 5). (**I**) Isolated CD8+ T-cells adjacent to the dentate gyrus of the hippocampus (case 4). Scale bars: (**A**,**B**) (upper panel), (**C**,**G**) 50 μm; (**B**) lower panel, (**D**,**H**) 20 μm; (**E**) ×0.7; (**F**) 100 μm; (**I**) 10 μm.

**Figure 2:**
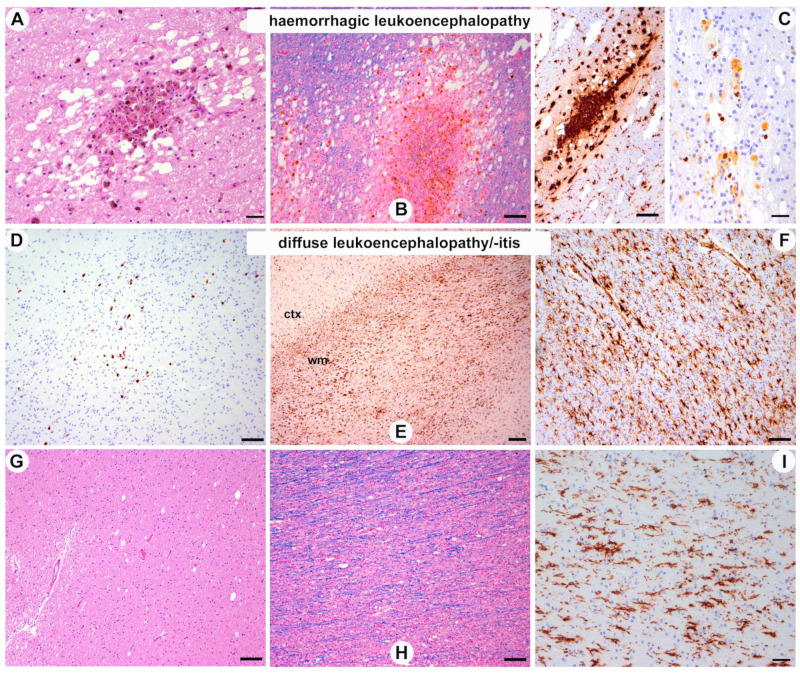
White matter pathology. (**A**–**C**) Multiple subacute hemorrhagic lesions throughout the white matter with hemosiderin-laden macrophages, surrounding Waller degeneration with axonal and myelin vacuolation ((**B**), luxol fast blue), proliferation of macrophages and activated microglia ((**C**), left panel anti-HLA-DR immunohistochemistry) and isolated CD8+ T-lymphocytes ((**C**), right panel; case 4). (**D**) Focal cellular infiltrates of CD8+ T-lymphocytes in the white matter (case 2) and prominent diffuse microglial activation, particularly in the white matter, independently of T-cell infiltrates ((**E**,**F**), anti-HLA-DR immunohistochemistry (ctx: cortex; wm: white matter). (**G**–**I**) mild to moderate diffuse vacuolation of white matter, better identified on luxol-fast-blue-stained sections, without signs of demyelination (**H**) and diffuse microglial activation, accentuated at the perivascular spaces ((**I**), anti-HLA-DR immunohistochemistry) ((**G**–**I**), case 5). Scale bars: (**A**,**C**) right panel, (**I**) 20 μm; (**B**,**C**) left panel, (**D**,**F**–**H**) 50 μm; (E) 100 μm.

**Figure 3:**
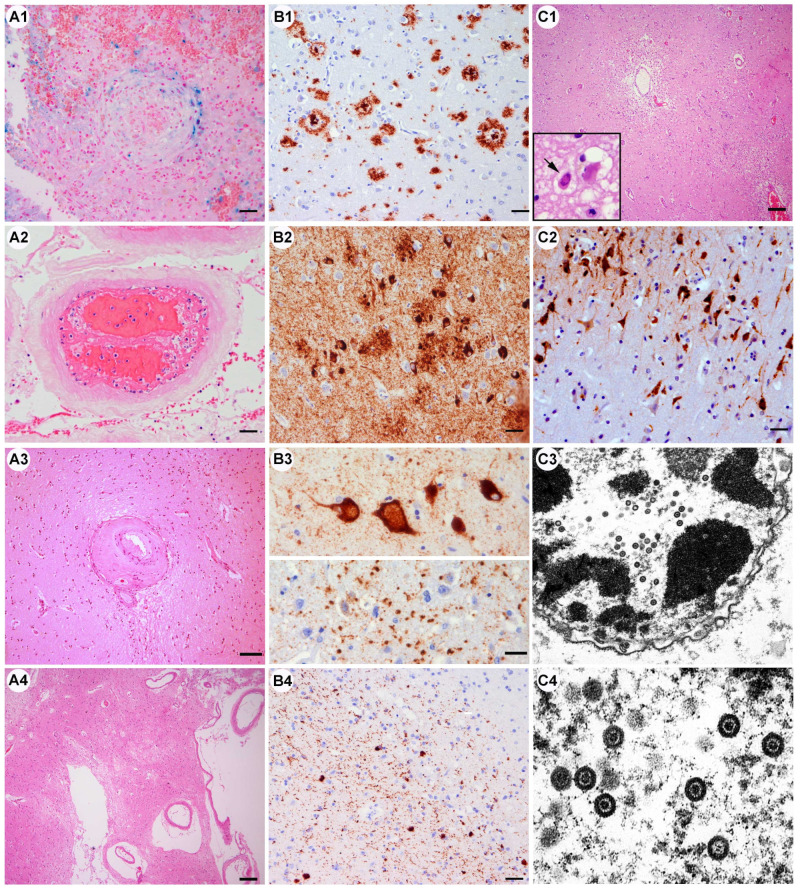
Other conditions. (**A1**–**A4**) Alteration of the vessel wall and vascular lumen. (**A1**) Vascular occlusion surrounded by fresh and old blood remnants (Pearl’s blue) and reactive tissue changes with gliosis, microglia, and macrophages (case 26). (**A2**) Early and partial fibrin clot in a meningeal vessel with entrapped leukocytes and erythrocytes. (**A3**) Eccentric thickening and fibrosis of small sized arterioles and perivascular tissue rarefaction (case 25) characteristic of small vessel disease that occurs in patients with chronic vascular risk factors. (**A4**) Prominent perivascular tissue rarefaction status cribosus of the basal ganglia (case 1) (all HE-stained sections). Scale bars: (**A1**,**A2**) 20 μm; (**A3**) 50 μm; (**A4**) 100 μm. (**B1**–**B4**) Pre-existing neurodegenerative pathology. (**B1**) Alzheimer’s disease neuropathological change. ßA4-amyloid deposits in brain parenchyma in form of compact and diffuse plaques (case 1) and within the wall of leptomeningeal and cortical vessels (inset, amyloid angiopathy; case 3). (**B2**) Tau-positive neurofibrillary pathology with abundant fine neuropil threads, intraneuronal neurofibrillary tangles, and dystrophic neurites surrounding amyloid plaques (case 1). (**B3**) Tau positive argyrophilic grain pathology with several enlarged “ballooned” neurons in the amygdala (upper panel) and grain-like structures in the neuropil (lower panel; case 25). (**B4**) Alpha-synuclein immunoreactive Lewy bodies and Lewy neurites in the olfactory bulb (case 4). Scale bars: (**B1**,**B2**,**B4**) 20 μm; (**B3**) 10 μm. (**C1**–**C4**) Concomitant HSV-1 infection. (C1) Diffuse hypoxic cortical damage with tissue pallor, endothelial activation, and diffuse inflammatory infiltrates. Inset: intranuclear neuronal inclusion. (**C2**) Immunohistochemistry shows abundant HSV1 viral antigens in cortical neurons. (**C3**,**C4**) Ultrastructural identification of neuronal intranuclear viral particles with typical morphology of Herpes simplex. (case #32). Scale bars: (**C1**) 100 μm (**C2**) 20 μm; (**C3**) original magnification ×30,000, (**C4**) ×80,000.

A summary of the results is presented in Table 2, Table 3, Table 4 and Table 5 and Supplementary Tables S1 and S2, as well as in Figure 1, Figure 2 and Figure 3.

### 3.1. Global Findings

All cases (case #1 to case #32) showed signs of diffuse edema with pericellular and perivascular tissue rarefaction. This was observed at different degrees of severity ranging from mild to severe. There was a spectrum of pathologies that ranged from a complete absence of focal lesions to acute ischemic infarcts, acute hemorrhages, single vascular thromboses, mild olfactory and/or cranial nerve neuritis, or nodular brainstem encephalitis. No signs of obvious vascular or meningeal inflammation were identified (see Table 2, Table 3, Table 4 and Table 5). All cases showed a variable degree of microglial activation, which was predominantly and diffusely affecting the white matter.

### 3.2. Specific Alterations

#### 3.2.1. Inflammatory Changes (Figure 1)

A: Olfactory system: eight patients showed mild CD8-positive T lymphocyte-dominated inflammatory infiltrates along the olfactory bulb and tract (cases #2, #3, #5, #6, #7, #12, #21, #22). In one patient, mild infiltrates were also noted within the entorhinal area adjacent to the hippocampus and amygdala (case #1). These infiltrates were accompanied by relatively marked microglial activation in both grey and white matter. All other cases showed diffuse, unspecific microglial activation but no obvious inflammatory nodules (Figure 1A,D,G). In H1N1-infected cases, olfactory bulb was unfortunately not available for analysis, but entorhinal areas also showed relatively prominent microglial activation in 2 of 3 cases (cases #1 and #2), one of them with small microglial nodules (Table 2, case #2).

B: (Micronodular) brain stem encephalitis: seven cases showed mild (cases #2, #6, #12, #16, #19, #20, #21, #32) and three cases moderate (cases #3, #4, #5) inflammatory nodules in the midbrain, pons, and/or medulla oblongata. The motor nucleus of the vagal nerve and its emergence from the brainstem was involved in most cases. The cellular infiltrates were mainly composed by CD8-positive T lymphocytes and microglia/macrophages (Figure 1B–D). No CD20- or CD79A-positive B-cells were identified in any brain region in the parenchyma, only isolated intravascular or isolated perivascular cells were seen in single cases. The distribution of pathology was comparable to that observed in three brains of H1N1-infected patients (Table 2). The spinal cord was analyzed in 10 cases, and no signs of myelitis were identified. No signs of meningeal inflammation were detected in any case.

C: Cranial nerve neuritis: two patients with brainstem inflammation (patient #2 and #5, Table 2) also showed moderate inflammatory infiltrates along the emerging 10th cranial nerve. This infiltrate was also composed by CD8-positive T-cells and macrophages without B-cells (Figure 1C,F).

We additionally analyzed the peripheral nerves (n. medianus) of 10 patients. We found no signs of neuritis, perineuritis, endotheliitis, vasculitis, or of demyelination suggestive of GBS/CIDP. Only one elderly patient showed a moderate axonal neuropathy. Spinal ganglia were not available.

Moreover, we investigated the formalin-fixed skeletal muscle of the forearm of 10 cases and found no signs of myositis, endotheliitis, vasculitis, necrotizing myopathy, or metabolic damage. Only two patients showed a tendency to type 2 fiber atrophy by immunohistochemistry (cases #17, #29), which could be attributed to the prolonged immobilization at the intensive care unit.

D: Co-occurring infections: one patient (case #32) with prior immunosuppression due to treated breast cancer presented an acute herpes simplex encephalitis (HSV1) in the context of severe respiratory distress due to bilateral SARS-CoV-2 pneumonia (Figure 3C1–C4).

Although there were no major differences in respect to inflammatory changes between patients who died of SARS-CoV2-related pneumonia and those with COVID-19 who died of other conditions, patients with longer intensive care and septic state had, in general, more perivascular CD8-positive cells and diffuse microglial activation than patients with shorter disease duration and acute death, caused by myocardial complications or the pulmonary embolism. We could not identify a specific immunoreactivity for SARS-CoV-2 antigens.

**Table 2:** Distribution of inflammatory infiltrates in selected brain areas in SARS-CoV-2 and H1N1 infection (0 = absent, i = isolated, + = mild, ++ = moderate, +++ abundant; for scoring details please see Section 2).

	Olfactory Bulb			Hippocampus			Cortical Areas Global			Basal Ganglia Global			Medulla Oblongata			Cerebellum Global		
	CD8	HLA-DR	CD20	CD8	HLA-DR	CD20	CD8	HLA-DR	CD20	CD8	HLA-DR	CD20	CD8	HLA-DR	CD20	CD8	HLA-DR	CD20
SARS-CoV-2	Perivascular/Parenchyma	Perivascular/Parenchyma	Perivascular/Parenchyma	Perivascular/Parenchyma	Perivascular/Parenchyma	Perivascular/Parenchyma	Perivascular/Parenchyma	Perivascular/Parenchyma	Perivascular/Parenchyma	Perivascular/Parenchyma	Perivascular/Parenchyma	Perivascular/Parenchyma	Perivascular/Parenchyma	Perivascular/Parenchyma	Perivascular/Parenchyma	Perivascular/Parenchyma	Perivascular/Parenchyma	Perivascular/Parenchyma
Case 1	na	na	na	+/0	+++/+++	0/0	+/0	++/++	0/0	+/0	++/++	0/0	na	na	na	na	na	na
Case 2	+/+	++/++	0/0	++/i	+++/++	0/0	++/0	+++/+++	i/0	+/0	++/++	0/0	++/+	+++/+++	i/0	++/i	+++/++	0/0
Case 3	+/++	++/++	0/0	+/0	++/++	0/0	+/0	++/++	0/0	+/0	++/+	0/0	++/++	++/++	0/0	++/+	++/++	0/0
Case 4	i/0	+++/+++	i/0	+/+	+++/++	i/0	+/0	+++/+++	i/0	+/0	+++/+++	i/0	+/++	+++/+++	0/0	+/0	+++/+++	0/0
Case 5	+/++	+++/+++	0/0	+/0	+++/++	0/0	+/0	+++/++	0/0	+/0	+++/++	0/0	+/++	+++/+++	+/0	+/0	+++/++	0/0
Case 6	+/+	++/++	0/0	+/0	+++/++	0/0	+/0	+++/++	0/0	+/0	++/++	0/0	++/++	+++/++	0/0	+/0	+++/++	0/0
Case 7	+/+	++/++	0/0	i/0	++/++	0/0	+/0	++/++	0/0	+/0	+/+	0/0	++/++	+++/++	i/0	+/0	++/++	0/0
Case 8	i/i	+/+	0/0	+/0	+/+	0/0	+/0	+/+	0/0	+/0	+/+	0/0	+/0	+/+	0/0	+/0	+/+	0/0
Case 9	i/0	++/++	0/0	i/0	+++/++	0/0	i/0	++/++	0/0	i/0	++/++	0/0	i/0	+++/+++	0/0	i/0	++/++	0/0
Case 10	na	na	0/0	+/0	+/+	0/0	+/0	+/+	0/0	+/0	+/+	0/0	+/0	+/+	0/0	+/0	+/+	0/0
Case 11	+/0	++/+	0/0	+/0	+/+	0/0	+/0	++/+	i/0	+/0	+/+	0/0	+/0	++/+	0/0	+/0	++/+	0/0
Case 12	+/+	++/++	0/0	+/0	+++/++	0/0	+/0	+++/+++	i/0	+/0	++/++	0/0	+/+	++/++	0/0	+/0	+++/+++	0/0
Case 13	+/0	+/+	0/0	+/0	+/+	0/0	+/0	+/+	0/0	+/0	+/+	0/0	+/0	+/+	0/0	+/0	+/+	0/0
Case 14	i/0	++/++	0/0	+/0	++/++ *	0/0	+/0	++/++	0/0	+/0	++/++	0/0	+/0	+++/+++	0/0	+/0	++/++	0/0
Case 15	i/0	++/++	0/0	+/0	++/++	0/0	+/0	++/++	0/0	+/0	++/++	0/0	+/0	++/++	0/0	+/0	++/++	0/0
Case 16	i/0	++/++	0/0	+/0	++/++	0/0	+/0	++/+	0/0	+/0	++/++	0/0	+/0	++/+++	0/0	+/0	++/++	0/0
Case 17	i/0	++/++	0/0	i/0	++/++	0/0	i/0	++/++	0/0	i/0	++/++	0/0	i/0	++/++	0/0	i/0	++/++	0/0
Case 18	i/0	++/++	0/0	+/0	+/+	0/0	+/0	+/+	0/0	+/0	+/+	i/0	+/0	++/++	0/0	+/0	++/++	0/0
Case 19	+/0	++/++	i/0	+/0	+++/++	i/0	+/0	+/+	0/0	+/0	++/++	0/0	++/+	+++/+++	0/0	+/0	+++/++	0/0
Case 20	+/0	+/+	i/0	+/0	++/+	0/0	+/0	+/+	i/0	+/0	+/+	i/0	+/0	++/++	0/0	+/0	++/+	0/0
Case 21	+/+	++/++	0/0	+/0	+/+	0/0	+/0	+/+	0/0	+/0	+/+	0/0	++/++	++/++	0/0	+/0	+/+	0/0
Case 22	+/++	++/++	0/0	+/0	++/+	+/0	+/0	+/+	0/0	+/0	++/++	+/+	+/i	++/+	i/0	++/0	++/+	i/0
Case 23	i/0	++/++	0/0	+/0	++/+	+/0	+/0	++/++	0/0	+/0	++/++	0/0	+/0	++/++	0/0	+/0	++/++	0/0
Case 24	i/0	++/++	0/0	+/0	++/+	0/0	+/0	++/++	0/0	+/0	++/++	0/0	+/0	++/++	0/0	+/0	++/++	0/0
Case 25	+/0	++/++	0/0	+/0	+++/++	0/0	+/0	+++/+++	0/0	+/0	+++/+++	0/0	+/0	+++/+++	0/0	+/0	+++/+++	0/0
Case 26	i/0	+/+	0/0	i/0	++/+	0/0	i/0	+/+	0/0	+/0	++/++	0/0	+/0	++/++	i/0	+/0	++/++	0/0
Case 27	i/0	++/++	0/0	i/0	++/++	0/0	i/0	+/+	0/0	i/0	++/++	0/0	+/0	++/++	0/0	+/0	++/++	0/0
Case 28	+/0	++/++	0/0	+/0	++/++	0/0	+/0	++/+	0/0	+/0	++/++	0/0	i/0	++/++	0/0	i/0	++/++	0/0
Case 29	i/0	++/++	0/0	+/0	++/+	0/0	i/0	++/++	0/0	i/0	++/++	0/0	i/0	++/++	0/0	i/0	++/++	0/0
Case 30	+/i	++/++	0/0	i/0	+++/+++	0/0	i/0	+++/+++	0/0	i/0	+++/+++	0/0	+/+	+++/+++	0/0	+/+	+++/+++	0/0
Case 31	i/0	+/++	0/0	i/0	++/+	0/0	i/0	+/+	0/0	i/0	+/+	0/0	i/0	++/++	0/0	i/0	++/+	0/0
Case 32	+/+	++/++	0/0	++/++	+++/+++	0/0	++/+	++/++	0/0	++/+	+++/+++	0/0	+/+	+++/+++	0/0	+/0	++/++	0/0
**H1N1**																		
Case 1	na	na	na	+/0	++/+	0/0	i/0	++/+	0/0	+/0	+/+	0/0	+/0	+/++	0/0	i/0	+/+	0/0
Case 2	na	na	na	+/0	+/++	0/0	i/0	+/++	0/0	+/0	++/++	0/0	+/0	++/++	0/0	+/0	++/++	0/0
Case 3	na	na	na	+/0	+/+	0/0	i/0	+/+	0/0	+/0	+/+	0/0	+/i	+/++	0/0	i/0	+/+	0/0

* around ßA4-amyloid plaques.

#### 3.2.2. White Matter Pathology (Figure 2)

A: Hemorrhagic leukoencephalopathy: one patient (case #4) showed multiple subacute white matter hemorrhages that were distributed throughout the supratentorial and infratentorial white matter and affected all cerebral lobes, the basal ganglia, brainstem, and cerebellum. The lesions showed a peripheral rim of hemosiderin pigment and central red blood cells in different stages of degradation (Figure 2A–C). These lesions were mostly associated with white matter vessels, but without signs of vasculitis, fibrinoid necrosis of the vascular wall, or thrombosis.

B: Leukoencephalitis: in one patient with olfactory neuritis and brain stem encephalitis, we further identified the mild foci of CD8-positive lymphocytes diffusely distributed within the white matter (Figure 2D–F, case #2 Table 2); however, these were identified without signs of selective demyelination.

C: Diffuse white matter damage: this was a nearly constant feature in all cases and ranged from subtle changes in the LFB stain to the formation of relatively prominent white matter vacuoles (Figure 2D–I), likely representing intramyelinic oedema. These changes were more prominent in posterior, parieto-occipital areas. Moreover, there was a diffuse, white matter predominant microglial activation with clear perivascular accentuation (Figure 2E,F,I, Table 3), which also involved the corpus callosum and the anterior commissure, and was associated with a perivascular accumulation of CD8-positive lymphocytes. No obvious phagocytosis of perivascular myelin such as in acute demyelinating encephalomyelitis (ADEM) was observed. Moreover, we could not identify demyelinating plaques in the white matter or cortical demyelination. No prominent white matter gliosis was detected. Axons were comparatively well preserved.

**Table 3:** Global distribution of inflammatory cells in supra- and infratentorial white matter (0 = absent, i = isolated, + = mild, ++ = moderate, +++ abundant; for scoring details please see Section 2).

	White Matter Supratentorial			White Matter Cerebellum		
	CD8	HLA-DR	CD20	CD8	HLA-DR	CD20
	Perivascular/Parenchyma	Perivascular/Parenchyma	Perivascular/Parenchyma	Perivascular/Parenchyma	Perivascular/Parenchyma	Perivascular/Parenchyma
Case 1	+/0	++/+++	0/0	na	na	na
Case 2	+/++	++/+++	0/0	++/i	+++/++	0/0
Case 3	+/0	++/+++	0/0	++/+	++/++	0/0
Case 4	+/0	++/+++	i/0	+/0	+++/+++	0/0
Case 5	+/0	+++/++	0/0	+/0	+++/++	0/0
Case 6	i/0	++/+++	0/0	+/0	+++/++	0/0
Case 7	+/0	++/++	0/0	+/0	++/++	0/0
Case 8	+/0	+/+	0/0	+/0	+/+	0/0
Case 9	i/0	++/++	0/0	i/0	++/++	0/0
Case 10	+/0	+/+	0/0	+/0	+/+	0/0
Case 11	+/0	+/+	0/0	+/0	++/+	0/0
Case 12	+/0	++/+++	0/0	+/0	+++/+++	0/0
Case 13	+/0	+/+	0/0	+/0	+/+	0/0
Case 14	+/0	++/++	0/0	+/0	++/++	0/0
Case 15	+/0	++/++	0/0	+/0	++/++	0/0
Case 16	+/0	++/++	0/0	+/0	++/++	0/0
Case 17	i/0	++/++	0/0	i/0	++/++	0/0
Case 18	+/0	++/++	0/0	+/0	++/++	0/0
Case 19	+/0	++/++	0/0	+/0	+++/++	0/0
Case 20	+/0	++/+	i/0	+/0	++/+	0/0
Case 21	+/0	++/++	0/0	+/0	+/+	0/0
Case 22	+/0	++/+	0/0	++/0	++/+	i/0
Case 23	+/0	++/++	0/0	+/0	++/++	0/0
Case 24	+/0	++/++	0/0	+/0	++/++	0/0
Case 25	+/0	+++/++	0/0	+/0	+++/+++	0/0
Case 26	+/0	++/+	0/0	+/0	++/++	0/0
Case 27	+/0	++/+	0/0	+/0	++/++	0/0
Case 28	+/0	++/+	0/0	i/0	++/++	0/0
Case 29	i/0	++/++	0/0	i/0	++/++	0/0
Case 30	i/0	+++/+++	0/0	+/+	+++/+++	0/0
Case 31	i/0	+/+	0/0	i/0	++/+	0/0
Case 32	+/0	+++/+++	0/0	+/0	++/++	0/0

#### 3.2.3. Vascular Pathology (Figure 3A, Table 4 and Table 5)

A: Vascular thrombosis: four cases: venous and arterial thrombosis was identified in a patient who also had a sinus vein thrombosis in the context of a generalized thrombotic event (case #9). One patient showed small arterial thrombosis in a meningeal vessel (case #19, Figure 3A2) and another a fibrin clot in the dural sinus (case #25). In case #26, we observed hemorrhagic lesions around a centrally located fresh venous thrombus in the frontal cortex. In the temporo-basal cortex, in an area of an apparently old traumatic injury, we found an occluded vessel with bleeding and hemosiderin deposits (Figure 3A1). The thrombotic events were associated with acute ischemic or hemorrhagic lesions.

We frequently found stagnant vessels with abundant entrapped leukocytes throughout most of the cases of our series, without the direct attachment of CD8-positive T-cells to endothelial cells, thrombosis, the disruption of the endothelial cell layer, and no signs of endotheliitis or vasculitis. This phenomenon is frequently observed in postmortem brains in the context of diffuse stagnation and sepsis, independent of the cause. The adherence of some neutrophils on the vessel wall should therefore not be considered a specific inflammation of the endothelium.

B: Diffuse hypoxic–ischemic damage: this type of damage was observed in several patients, either in relation to the venous or arterial thrombosis or in the context of a cardiac dysfunction (Table 5). Moreover, most patients suffered from severe pneumonia, a frequent cause of brain hypoxia. Hippocampus CA4 and CA1 sectors, thalamic neurons, cerebellar Purkinje cells, and dentate nucleus neurons appeared to be most vulnerable. Areas of acute or subacute cortical laminar necrosis were also identified (cases #6, #12, #13, #20, #25).

C: Acute hemorrhages: one patient suffered acute cerebellar bleeding in the context of arterial hypertension and dialysis (patient #23, Table 2).

D: Small vessel disease: most of the patients over 60 had pre-existing vascular risk factors (see Table 1). The brain vessels in the white matter and basal ganglia showed diffuse fibrosis of the wall, perivascular tissue rarefaction, and frequent yellowish perivasal lipopigment. In the basal ganglia cribriform, the change was moderate or prominent in nine cases (30%, cases #1, #7, #8, #10, #11, #14, #15, #28, #29)) (Figure 3A3,A4), while small lacunar and cerebellar infarcts were detected in five (16%, cases #1, #10, #11, #13, #29). Diffuse white matter rarefaction, particularly around fibrotic vessels and around the lateral ventricles, was also observed in these cases.

E: Large territorial infarcts: these were only observed in single patients who had focal neurological symptoms or seizures and were associated with vascular thrombosis (cases #25, #26). Neurologically asymptomatic patients did not show “silent” infarcts.

F: Incidental findings: in one patient, we identified a small vascular malformation—a cavernoma—in the frontal white matter (case #5).

#### 3.2.4. Pre-Existing Neurodegenerative Pathology (Figure 3B, Table 4)

A: Alzheimer’s disease neuropathological change: the combination of tau-positive neurofibrillary pathology and ßA4-amyloid/neuritic plaques +/− amyloid angiopathy was identified in 10 patients (32%) (Figure 3B1,B2), most of them aged over 70 years (mean 79, median 80, range 62–97) (cases #1,#3,#5,#10,#14, #17, #18, #26, #27, #28), but not in younger patients. The alterations consisted of neurofibrillary tangles and neuropil threads in the transentorhinal region and the limbic system (Braak stages I–IV), and they affected in some patients also neocortical areas (stages V and VI). ß-Amyloid pathology was in the form of diffuse and compact/cored amyloid plaques in mild, moderate, or abundant densities (CERAD plaque scores A, B, C, respectively) throughout the cortical areas, the limbic system, basal ganglia, brainstem, and cerebellum (Thal amyloid phases 1–5; severity scores ranging from A1, B1, and C1 to A3, B3, and C3 (Supplementary Table S2). Amyloid angiopathy was prominent in two of four cases. There was no increased inflammatory reaction around amyloid plaques and there were no cases of ß-amyloid-related angiitis (ABRA). Primary age-related tauopathy (PART) without significant ßA4-plaque pathology was identified in five patients below 70 years of age (16%, cases #2, #4, #11, #15, #16).

B: Argyrophilic grain disease: this limbic-predominant four-repeat tau pathology was observed in six patients (aged 64 to 86; cases #6, #8, #18, #25, #26, #28). Interestingly, the highest burden of tau pathology was identified in the youngest patient (Figure 3B3, case #25).

C: Lewy body pathology: alpha-synuclein immunoreactive Lewy bodies and Lewy neurites were identified in three patients in early brainstem or olfactory stages (cases #4, #10, #11), mainly affecting the dorsal motor nucleus of the vagal nerve, the olfactory bulb and tract, and the olfactory area around the amygdala. As these patients had not been clinically diagnosed as having Parkinson’s disease, they most likely represent an “incidental” Lewy body pathology (Figure 3B4).

D: TDP43 proteinopathy: two elderly patients (88 and 86 years, case #1 and #26) showed pTDP-43 protein aggregates in the limbic system representing LATE (limbic age-related TDP43 encephalopathy), with neither hippocampal sclerosis nor signs of motor neuron disease or extensive fronto-temporal involvement.

E: Prion disease: not a single case of (co-)incidental Creutzfeldt–Jakob disease or other type of prion disease was observed.

#### 3.2.5. Other

One patient suffered from a traumatic subdural hemorrhage and severe brain contusion that led to death (case #29); their death was not causally linked to COVID-19.

**Table 4:** Summary of neuropathological findings.

Pathology (n = 32)	n° of Cases (%)
Vascular	
Small vessel disease, pre-exisiting	11 (34%)
Large territorial infarct	7 (22%)
Vascular thrombosis	4 (12.5%)
Vasculitis/endotheliitis	0
Diffuse hypoxic–ischemic damage	13 (40%)
Acute hemorrhage	1 (3%)
Inflammation	
Olfactory neuritis	8 (25%)
Brainstem encephalitis micronodular	10 (31%)
Cranial nerve neuritis	2 (6%)
Encephalitis	0
Myelitis	0
Meningitis/ependymitis/plexitis	0
Peripheral neuritis	0
Myositis	0
Vasculitis/endotheliitis	0
White matter pathology	
Hemorrhagic leukoencephalopathy	1 (3%)
Diffuse leukoencephalopathy/microglial activation	32 (100%)
ADEM	0
Neurodegenerative changes preexisting	
ADNC	10 (32%)
PART	5 (16%)
ARTAG	2 (6%)
Lewy bodies	3 (9%)
TDP-43 proteinopathy	2 (6%)
Argyrophilic grain disease	6 (18%)
Other	
Fulminant Herpes simplex encephalitis	1 (3%)
Acute traumatic brain injury	1 (3%)

**Table 5:** Individual neuropathological diagnoses.

Case	Age	Sex	Inflammation	Vascular Pathology/Other	Neurodeg Pathology
Case 1	88	F	-	SVD, lacunar infarct	ADNC A2B3C2, LATE
Case 2	79	M	Olfactory neuritis, micronodular brainstem encephalitis, cranial nerve neuritis	Patchy acute hypoxic–ischemic neuronal damage	PART II, ARTAG
Case 3	80	F	Olfactory neuritis, micronodular brainstem encephalitis	-	ADNC A3B3C2 + CAA
Case 4	62	F	micronodular brainstem encephalitis	Hemorrhagic leukoencephalopathy	iLBD 3, PART II
Case 5	69	M	Olfactory neuritis, micronodular brainstem encephalitis, cranial nerve neuritis	Incidental cavernoma frontal	ADNC A3B2C2 + CAA
Case 6	79	M	Olfactory neuritis, micronodular brainstem encephalitis	Laminar occipital cortical necrosis older bilat (Morel)	AgD II
Case 7	58	M	Olfactory neuritis	SVD	-
Case 8	71	F	-	Patchy acute hypoxic–ischemic neuronal damage, SVD	AgD I
Case 9	58	M	-	Severe diffuse posthypoxic–postischemic pan encephalopathy with dural sinus thrombosis and meningeal vessel thrombosis; brainstem hemorrhage (Duret)	ADNC A1B0C0
Case 10	80	M	-	Old infarct cerebellum, SVD	LBD 4, ADNC A2B1C2
Case 11	80	F	-	Old lacunar infarct pons, SVD, patchy acute hypoxic–ischemic neuronal damage	iLBD 1, PART III
Case 12	66	M	Olfactory neuritis, micronodular brainstem encephalitis	Acute cortical laminar necrosis occipital and parietal	-
Case 13	54	M	-	Subacute infarct cerebellum	-
Case 14	97	F	-	SVD	ADNC A3B3C3 + CAA
Case 15	71	F	-	SVD, patchy acute hypoxic–ischemic neuronal damage	PART II
Case 16	57	F	Micronodular brainstem encephalitis	Patchy acute hypoxic–ischemic neuronal damage	PART II
Case 17	81	M	-	Patchy acute hypoxic–ischemic neuronal damage	ADNC A3B1C1
Case 18	73	M	-	-	ADNC A1B1C1, AgD I
Case 19	64	M	Micronodular brainstem encephalitis	Small thrombus meningeal vessels frontal	ARTAG
Case 20	66	M	Micronodular brainstem encephalitis	Acute partly hemorrhagic cortical infarcts cingulum and parietal ctx	-
Case 21	38	M	Olfactory neuritis, micronodular brainstem encephalitis	Diffuse acute hypoxic–ischemic neuronal damage, CO intoxication	-
Case 22	34	M	Olfactory neuritis	Patchy acute hypoxic–ischemic neuronal damage	-
Case 23	72	M	-	Old SDH and SAB, patchy acute hypoxic–ischemic neuronal damage	-
Case 24	52	F	-	Perivascular fibrosis (systemic sclerosis known)	-
Case 25	64	M	Mild hypothalamus	Multiple bilateral old cortical laminar necrosis and cerebellar necrosis; sinus vein thrombosis	AgD III
Case 26	86	M	-	Hemorrhagic infarcts frontal and frontobasal, vascular thrombosis	AgD I, LATE, ADNC A2B1C1
Case 27	62	F	-	Severe venous congestion and SAB occipital	ADNC A2B0C1 + CAA
Case 28	82	F	-	SVD, acute traumatic SDH + SAB + acute hypoxic damage + diffuse axonal injury	AgD II, ADNC A3B2C2
Case 29	67	F	-	Old thalamic lacunar infarct, SVD, metabolic gliosis	ADNC A1B0C0
Case 30	49	M	-	Multiple subacute infarcts cingulum, amygdala, occipitomental, pons, medulla dorsolateral, cerebellum; Wernicke-like changes (alcohol abuse)	-
Case 31	21	M	-	Patchy acute hypoxic–ischemic neuronal damage	-
Case 32	57	F	HSV-related encephalitis	Red neurons in encephalitis regions	

#### 3.2.6. H1N1-Infected Cases (Table 2)

The three patients who died of acute respiratory distress syndrome after H1N1 infection had similar neuropathological changes to those described here for SARS-CoV-2 (see also Table 2). Case #1 (f, 57) and Case #2 (m, 57) showed a disseminated microangiopathy with fibrinoid necrosis of several vessels with disseminated microhemorrhages in white matter hemorrhagic leukoencephalopathy, associated with moderate hypoxic–ischemic damage and diffuse edema, the latter also present in case #3. Moreover, disseminated fungal microabscesses (fungal pneumonia and sepsis, case #2) and a fungal septic necrotic focus in the occipital white matter (septic shock, case #1) were identified. Both cases also had brainstem microglial nodules and case #3 diffuse and brainstem predominant microglial activation. Patient #2 also showed a mild metabolic encephalopathy with increased Alzheimer type II astrocytes. No viral antigens could be demonstrated by PCR in any of the three cases in brain tissue.

## 4. Discussion

We present the neuropathological findings in a series of SARS-CoV-2-infected patients who died during the pandemic in 2020–2021 in Austria; two thirds of these patients had severe COVID-19 and received ICU treatment. We observed that one common neuropathological feature is the development of a diffuse white matter damage with intramyelinic edema and prominent diffuse and perivascular-accentuated microglial activation associated with the mild accumulation of perivascular CD8-positive T-cells and comparatively less axonal damage. The intensity of these alterations varied between cases, but it was globally moderate to severe and also included single cases with prominent vacuolar leukoencephalopathy, partly reminiscent of posterior reversible encephalopathy syndrome (PRES-like) [22], and one case of a micro-hemorrhagic HURST-like leukoencephalopathy [23]. The latter was also observed in one of three patients with H1N1 influenza infection from 2009 to 2010 and could be also related to disseminated intravascular coagulation, among other factors.

These changes seemed to be unrelated to the presence of acute/focal lesions and were accentuated in the parieto-occipital regions. Diffuse white matter damage is a non-specific finding and may be caused by several afflictions. Aside from inherited conditions causing, for example, hereditary leukodystrophies or CADASIL, acquired toxic or metabolic derangements may also alter the myelin sheath. We propose that a (severe) SARS-CoV-2 infection as in most of our cases may induce myelin damage not by the virus itself, but by a combination of an excessive cytokine release by the immune system and a metabolic/hydroelectrolytic derangement caused by the functional damage of vital organs and/or toxic damage by poly-medication (e.g., anticoagulation during extracorporeal membrane oxygenation).

During the acute phase of the disease, around 34% of patients experienced impaired memory, concentration, or attention [24]. Some recent studies have examined the immune cell profile in the CSF of patients with “Neuro-COVID-19” by single-cell sequencing [25,26] and found signs of local immune overactivation with a broad clonal T-cell expansion and reduced interferon response [25]. Another recent study performed a multidimensional characterization of immune mediators in the CSF and plasma of patients belonging to different Neuro-COVID-19 severity classes and identified a distinctive set of CSF and plasma mediators associated with blood–brain barrier (BBB) impairment, elevated microglia activation markers, and a polyclonal B-cell response targeting self-antigens and non-self-antigens underlying post-acute COVID-19 syndrome [27]. Altered BBB biomarkers have been also described in infected patients with neurological complications by other authors [28]. These results from other studies support the existence of immune-mediated mechanisms in severe COVID-19 that may contribute to neurological alterations, and that there might be a compromised antiviral response [25,26,29]. While one group did not observe an expansion of B-cells in CSF, findings that are also in line with our postmortem study where a B-cell component was nearly absent, others did. These differences between previous studies may have resulted from the analysis at different time points of the disease. Yet, another study analyzed several cyto/chemokines in the CSF and/or sera of patients with COVID-19 and neurological symptoms and found predominantly an alteration of the neurovascular unit rather than a prominent COVID-19-related neuroinflammatory profile [30]. Furthermore, a recent study performed in mice and humans observed a white matter-selective microglia reactivity after SARS-CoV-2 infection. The authors also observed a persistently impaired hippocampal neurogenesis, decreased oligodendrocyte counts, and signs of myelin loss in the context of increased CSF cytokines/chemokine levels, including CCL11 [31]. These findings were also detected after H1N1 influenza infection. These previous experimental findings do also support our postmortem findings of diffuse white matter microglial activation and likely intramyelinic edema.

In the post-illness stage, 15–80% of patients complained of cognitive dysfunction as part of a long-term clinical manifestation called post-COVID-19 syndrome [32]. This syndrome encompasses symptoms that persist after about 12 weeks of infection and include fatigue and concentration, attention, and memory deficits, among others. As white matter plays an important role in cognition [33], it might well be that acute and diffuse, probably multifactorial, white matter damage contributes to the development of post-COVID-19 cognitive dysfunction. In addition to the possible consequences of altered cytokine/chemokine levels, acute vascular pathologies, including thrombotic events, hypoxic/ischemic/hemorrhagic lesions in strategic brain areas, and/or the presence of pre-existing small vessel disease with/without leukoencephalopathy in patients with cardiovascular risk factors (altogether roughly observed in about one third of our cohort) may contribute to short-, mid-, or even long-term memory/attention problems in post-COVID-19 syndrome [34,35,36,37]. Additionally, important are underlying “preclinical” neurodegenerative morbidities in elderly people (e.g., Alzheimer type changes, limbic TDP-43 proteinopathy, premotor Lewy body pathology, argyrophilic grain pathology) might act as additional factors. These frequently develop in strategic limbic and brainstem nuclei (in our study, in about two thirds of patients, including patients in their late 50s/early 60s) and may also contribute to cognitive deficits after SARS-CoV-2 infection.

The reversibility of these changes will probably depend on the severity of the myelin damage and axonal integrity as well as on the host-dependent immune system, cognitive reserve, and several yet unknown factors. Of note, cognitive dysfunction has been also repeatedly reported after critical illness [38].

As we learnt from previous pandemics, such as the one caused by H1N1 virus in 1915–1930, long-term neurological consequences might appear even decades after the infection. A good example is linked to postencephalitic parkinsonism, a neurodegenerative tauopathy that developed years after influenza infection and an episode of encephalitis lethargica. In those cases, however, no viral antigens have yet been demonstrated in the brains [39]. It has been therefore postulated that an immunological process would be more likely the trigger of neurodegeneration rather than the virus per se. Therefore, it will be of utmost importance to perform future neuropathological studies in patients who survive COVID-19 and die years later of another condition, to investigate whether there are any sequelae, including unusual neurodegenerative features. It will be also particularly important to perform well-designed and controlled morphometric studies.

We observed a mild inflammatory CD8-positive T-cell infiltration of the bulbus/tractus olfactorius in eight cases. In addition, we identified a mild to moderate micronodular brainstem encephalitis in ten patients. In two of those, a cranial nerve inflammation was also identified. It is a matter of debate whether these changes are directly related to the viral infection and contribute to some of the symptoms, including the prominent brainstem dysfunction and respiratory problems the patients present, or whether this is a consequence of severe, end-stage diseases [40,41,42,43]. As in some other studies [44,45], we could not identify a specific signal for SARS-CoV-2 antigens by conventional immunohistochemistry, but this does not exclude the presence of viral particles. Some authors have detected small viral sequences by RT-PCR and RNAseq in a small fraction of patients [46], but it is still unclear whether they represent true CNS infection. Further studies with other, more sensitive modalities are currently in progress. The micronodular brain stem encephalitis that we observed in about one third of the SARS-CoV-2-infected patients was comparable to that seen in the three H1N1-infected patients of our study, and might therefore not necessarily be a direct consequence of a SARS-CoV-2 infection itself. Microglial activation in the postmortem human brain is a frequent, non-specific finding and can be observed in a vast range of conditions, particularly in the context of critical illness, where an interplay of hypoxic–systemic–metabolic–infectious conditions generally contribute to death [17,47,48]. An alternative cause of these findings could be a reactivation of a latent viral infection of another type. This is frequently observed in autopsy series in the context of sepsis or severe terminal disease [49] and is not unique to SARS-CoV-2 infection. Interestingly, a single patient with chemotherapy-related immunosuppression developed a fulminant necrotizing herpes simplex virus encephalitis in the context of SARS and bilateral pneumonia. Moreover, two patients had a non-CNS reactivation of herpes simplex virus infection, two patients of Epstein–Barr virus, one patient suffered from aspergillosis, and one from mucormycosis. These co-morbid conditions might have contributed to patients’ immune response in the brain and underscores the importance of ruling out potential co-occurring infections. Per contra, patients were critically ill and received several medications, including antiviral, antibiotic, antimycotic, and anti-inflammatory drugs, including corticosteroids, which likely altered the inflammatory milieu in the brain, influencing the postmortem neuropathological findings, which is another note of caution for the interpretation of our results.

The limitations of the study include its retrospective nature, the analysis of postmortem brain tissue of a clinically heterogeneous patients group with different pre-existing conditions and risk factors, different clinical evolutions and treatment strategies, and the lack of patients with “post-COVID-19” syndrome.

In summary, our results support some previous neuropathological findings of an apparently multifactorial brain damage in the context of SARS-CoV-2 infection and are in line with recent experimental data on diffuse white matter damage, microglial activation, and cytokine release. At least, the alpha and/or delta SARS-CoV-2 variant that affected most of the patients of our study seem not to be what is classically considered a “neuro(no)tropic virus” in a strict sense [26,46,50,51]. Such neurotropism combines neuroinvasiveness, neurovirulence, and neuroreplication, the direct infection of neural cells/neurons by the virus and its replication there, causing an acute polioencephalitis-associated variable T-cell-mediated inflammation and neuronal loss, as known for well-established neurotropic viruses, e.g., flaviviruses [52]. In contrast, we believe that the prominent immunological reaction induced by the SARS-CoV-2 virus and subsequent cytokine boost, potential immunological mimicry of SARS-CoV-2 antibodies with neuronal/glial antigens [53], the septic condition, and the dysfunction of vital organs with subsequent hypoxic brain damage, mitochondrial/metabolic/hydroelectrolytic derangements, are all likely contributors to a diffuse myelin damage, as already observed in other viral diseases where a pathogenetic network co-orchestrates virus-specific as well as -unspecific factors, e.g., in HIV infection [54]. Such alterations may decompensate or accelerate pre-existing “silent” brain conditions, in particular, underlying subtle neurodegenerative pathologies, chronic vascular pathologies, and latent viral infections, that might alter synaptic and neurotransmitter functions in the short-, mid-, and long-term.

While those who died are not necessarily representative of the majority of people who survive and develop long COVID-19/post COVID-19 and cognitive sequelae, the observed white matter damage in postmortem studies might be an important contributing factor to the long-term effects of COVID-19 infection.

Whether new virus variants will change or broaden the spectrum of neurological complications remains unclear, but it is likely. More recent COVID-19 variants that are likely to have a lower mortality may perhaps have less neuropathological impact. Further interdisciplinary studies are essential, and neuropathological expertise and brain banks can provide a valuable resource of tissue-based information to further elucidate the neurological, and particularly the cognitive consequences/sequelae of a severe infection, with multiorgan involvement, as exemplified by SARS-CoV-2.

## Figures and Tables

**Table 1:** Basic clinical data.

	Age	Sex	Clinical Diagnosis	Cause of Death	Disease Duration	Days in ICU	Neurological Symptoms	Vascular Risk Factors	Additional co-Morbidities	Treatments	SARS-CoV-2 PCR Positivity
Case 1	88	Female	na	na	na	na	na	na	na	na	na *
Case 2	78	Male	COVID-19 pneumonia	Cardiovascular failure	56	48	Did not regain consciousness after stop of narcotics	Diabetes, hypertension, obesity, atrial fibrillation	Dilatative cardiomyopathy, arteriosclerosis	Remdesivir	blood, perianal, trachea, larynx
Case 3	79	Female	COVID-19 pneumonia	Pulmonary thrombosis, pulmonary bleeding, Sepsis	18	11	None	Hypertension, obesity	-	Kaletra, cortisone, 3 doses reconvalescent plasma, remdesivir	Larynx
Case 4	62	Female	COVID-19 pneumonia	Respiratory failure	na	63	Critical illness polyneuropathy/myopathy, reduced vigilance, EEG diffuse slowing	Atrial fibrillation, arterial hypertension	Suspicion of latent tuberculosis	Cortisone; positive EBV PCR- Ganciclovir	na *
Case 5	68	Male	COVID-19 pneumonia	Multi-organ failure	31	27	Agitation, confusion, aggression, psychosis	Diabetes, hypertension, obesity	Pulmonary emphysema, steatosis hepatis, arteriosclerosis	Kaletra, remdesivir, cortisone, Roactemra, 3 doses reconvalescent plasma	larynx, trachea
Case 6	78	Male	COVID-19 pneumonia	Multi-organ failure	28	25	Did not regain consciousness after stop of narcotics, anisocoria, meningismus, tetraparesis	Arterial hypertension, atrial fibrillation	Pulmonary emphysema, arteriosclerosis, chronic kidney injury, prostate hyperplasia, hyperuricemia	Reconvalescent plasma, Roactemra, dialysis	trachea, larynx
Case 7	57	Male	COVID-19 pneumonia	Respiratory failure	5	0	None	Arterial hypertension, obesity	Steatosis hepatis	None	naso-pharynx
Case 8	71	Female	COVID-19 pneumonia	Multi-organ failure secondary to respiratory failure due to pneumonia	10	4	na	Arteriosclerosis	Myotonic dystrophy type 2, steatosis hepatis, asthma	Cortisone, remdesivir	naso-pharynx
Case 9	58	Male	COVID-19 pneumonia	Sinus thrombosis	26	7	Seizures, coma	Chronic kidney failure, arteriosclerosis	Pulmonary emphysema	None	trachea, naso-pharynx
Case 10	80	Male	COVID-19 pneumonia	Respiratory failure	7	0	Anxiety disorder, somnolence due to psychopharmaceuticals	Arterial hypertension, arteriosclerosis, acute on chronic kidney disease	Humerus fracture left arm	Ceftriaxone, hydroxychloroquine, zinc	na *
Case 11	80	Female	COVID-19 pneumonia	Cardiorespiratory failure	3	3	None	Cardiac insufficiency (NYHA IV), diabetes, arterial hypertension, arteriosclerosis	-	None due to rapid cardiac deterioration	na *
Case 12	66	Male	COVID-19 pneumonia	Multi-organ failure, hypoxic encephalopathy	42	35	Impaired consciousness of unknown origin, potentially due to hypoxic encephalopathy	Arterial hypertension, hypercholesterinemia	Status post transient ischemic attack 2014	Cortisone, remdesivir; EBV, HSV and Aspergillus infection	na *
Case 13	54	Male	COVID-19 pneumonia, mucormycosis with pulmonary infarction	Respiratory failure	24	18	None	Obesity, arterial hypertension, hypertrophic left ventricle	Myelodysplastic syndrome, secondary acute myeloid leukemia with chemotherapy and complete hematologic remission, bone marrow hypoplasia, signs of dysplastic hematopoiesis in the peripheral blood	Tocilizumab	na *
Case 14	96	Female	Rib fractures, hemothorax, and COVID-19 pneumonia	Respiratory failure	na	na	na	Arterial hypertension, hypertensive cardiomyopathy, pulmonary hypertension	-	None	naso-pharynx
Case 15	71	Male	COVID-19 pneumonia	Multi-organ failure secondary to respiratory failure due to pneumonia with interstitial fibrosis	24	15	Ageusia, agitation	Arterial hypertension	Arteriosclerosis, steatosis hepatis, cholecystolithiasis	Cortisone, remdesivir	trachea, naso-pharynx, bronchial
Case 16	56	Female	COVID-19 pneumonia	Hemorrhagic shock after ECMO implantation	15	12	None	Diabetes, obesity	Status post thyroid carcinoma	Cortisone, remdesivir	naso-pharynx, trachea
Case 17	81	Male	Unclear, postoperative multi-organ failure	Multi-organ failure	na	1	None	Intermittent atrial fibrillation	IPMN of the pancreas	None	na *
Case 18	73	Male	COVID-19 pneumonia	Cardiogenic shock after ECMO implantation	16	11	None	Arterial hypertension, hypercholesterinemia	-	Cortisone, remdesivir	naso-pharynx, trachea
Case 19	63	Female	COVID-19 pneumonia	Respiratory failure	1	0	None	Arterial hypertension, obesity, arteriosclerosis	Chronic venous insufficiency, steatosis hepatis	None	naso-pharynx
Case 20	65	Male	COVID-19 pneumonia	Respiratory failure	36	26	Hemiparesis right side (onset 1 November 2021)	Obesity, sleep apnea syndrome, arterial hypertension, diabetes	Steatosis hepatis	Cortisone	naso-pharynx
Case 21	38	Male	Not applicable (death at home)	Carbon monoxide poisoning	na	0	na	None	-	None	naso-pharynx post mortem
Case 22	33	Male	Cardiac arrest	Cardiovascular failure	7	0	None	None	-	None	naso-pharynx
Case 23	71	Male	COVID-19 pneumonia	Multi-organ failure	71	57	None/prolonged weaning	Diabetes, arterial hypertension, PAOD, hyperlipidemia	Status post TIA	Cortisone	naso-pharynx
Case 24	52	Female	COVID-19 pneumonia	Cardiogenic shock due to myocarditis, death during new cannulation of ECMO	12	5	None	Obesity	Scleroderma	Cortisone	naso-pharynx
Case 25	64	Male	Sepsis	Multi-organ failure	na	34	Coma, therapy-resistant status epilepticus	Arteriosclerosis, arterial hypertension, obesity, chronic kidney disease, hypercholesterinemia, nicotine abuse	Alcohol abuse, steatosis hepatis, chronic pancreatitis, pulmonary edema and congestion, hypothyroidism, hyperuricemia, pericarditis	None	naso-pharynx
Case 26	86	Male	Cardiac arrest	Diffuse myocardial ischemia	na	0	Delirium	Arteriosclerosis, arterial hypertension	Prostate hyperplasia, status post meningioma resection left frontal lobe 9 days before death	Cortisone	naso-pharynx
Case 27	62	Female	COVID-19 pneumonia	Respiratory failure	14	4	na	None	-	Cortisone	naso-pharynx
Case 28	82	Female	Subdural hematoma	Subdural hematoma	8	0	Coma, anisocoria	Atrial fibrillation	Anxiety disorder	None	naso-pharynx
Case 29	67	Female	Not applicable (death at home)	Cardiovascular failure	na	0	na	Arterial hypertension, atrial fibrillation	Chronic kidney failure	None	naso-pharynx post mortem
Case 30	49	Male	Progressive stroke	Cardiovascular failure	61	12	Stroke with visual disturbance, gait disturbance, dysphagia, dysarthria, aphasia, agitation, reduced vigilance	Diabetes, arterial hypertension	Chronic kidney disease, schizoaffective disorder	Cortisone	naso-pharynx
Case 31	21	Male	Myocardial infarction	Acute myocardial infarction	na	0	Epileptic seizure	Obesity	Purpura Schönlein–Henoch	None	naso-pharynx
Case 32	57	Female	COVID-19 pneumonia	Cardiorespiratory failure	na	na	na	Arteriosclerosis	Invasive mammary carcinoma	na	na *

na *: all patients had PCR confirmed SARS-CoV-2 infection; in cases with na * exact sampling site for PCR was not available.

## Data Availability

Data are available upon reasonable request to the corresponding authors.

## Supplementary Materials

The following supporting information can be downloaded at: https://www.mdpi.com/article/10.3390/v15040908/s1, Table S1: List of primary antibodies; Table S2: Semiquantitative assessment and mapping of inflammatory infiltrates across the different brain regions.
