# Supplementary material for: Multifactorial White Matter Damage in the Acute Phase and Pre-Existing Conditions May Drive Cognitive Dysfunction after SARS-CoV-2 Infection: Neuropathology-Based Evidence
Source: Viruses. 2023 Mar 31;15(4):908. doi: 10.3390/v15040908 (PMC10144140; doi:10.3390/v15040908)
Supplement: Supplementary file 1 [file viruses-15-00908-s001.zip › Supplementary Table S1.pdf]

Supplementary Table S1: Antibodies used for immunohistochemistry

Antibody	Clone	Company	Dilution	positive control
CD8	clone C8/144B	DAKO, Glostrup, Denmark	1:100	lymph node
CD20	clone L26	DAKO, Glostrup, Denmark	1:400	lymph node
HLA-DR	clone CR3/43	DAKO, Glostrup, Denmark	1:400	brain infarction, ischaemic, postmortem brain tissue
ßA4	clone 6F/3D	DAKO, Glostrup, Denmark	1:100	Alzheimer's disease postmortem brain tissue
AT8	clone AT8, pS202/pT205	Thermo Scientific, Rockford, IL, USA	1:200	Alzheimer's disease postmortem brain tissue
a-synuclein	clone 5G4	Roboscreen, Leipzig, Germany	1:4,000	Parkinson's disease postmortem brain tissue
pTDP43	clone 11-9, pS409/410	Cosmo Bio, Tokyo, Japan	1:20,000	Fronto-temporal lobar degeneration postmortem brain tissue
p62	clone 3/p62 lck ligand	BD Transduction Laboratories, Franklin Lakes, NJ, USA	1:500	Alzheimer's disease postmortem brain tissue
MBP	clone BS188	Nordic BioSite AB, Täby, Sweden	1:200	control white matter postmortem brain tissue
Myosin slow	WB-MHCs	Novocastra, Leica Microsystems, Deer Park, IL, USA	1:50	muscle, quadriceps
Myosin fast	WB-MHCf	Novocastra, Leica Microsystems, Deer Park, IL, USA	1:50	muscle, quadriceps
SARS-COV2	rb Spike; ms N-protein	Sinobiological, Beijing, China	1:1000	postmortem lung of COVID19 pneumonia
