# Supplementary material for: Multifactorial White Matter Damage in the Acute Phase and Pre-Existing Conditions May Drive Cognitive Dysfunction after SARS-CoV-2 Infection: Neuropathology-Based Evidence
Source: Viruses. 2023 Mar 31;15(4):908. doi: 10.3390/v15040908 (PMC10144140; doi:10.3390/v15040908)
Supplement: Supplementary file 1 [file viruses-15-00908-s001.zip › Supplementary Table S2.pdf]

Supplementary Table S2: Mapping of inflammatory infiltrates in different brain areas

	olfactory bulb			ento/transentorhinal region			amygdala			hippocampus		
	CD8	HLA-DR	CD20	CD8	HLA-DR	CD20	CD8	HLA-DR	CD20	CD8	HLA-DR	CD20
	perivascular / parenchyma	perivascular / parenchyma	perivascular / parenchyma	perivascular / parenchyma	perivascular / parenchyma	perivascular / parenchyma	perivascular / parenchyma	perivascular / parenchyma	perivascular / parenchyma	perivascular / parenchyma	perivascular / parenchyma	perivascular / parenchyma
case 1	na	na	na	+ / +	+++/+++	0 / 0	+ / 0	+++/+++	0 / 0	+ / 0	+++ / +++	0 / 0
case 2	+/+	++ / ++	0 / 0	+ / 0	+++ / ++	i / 0	++ / +	+++/+++	i / 0	++ / i	+++ / ++	0 / 0
case 3	+ / ++	++ / ++	0 / 0	+ / 0	++ / +	0 / 0	+ / 0	++ / ++ **	0 / 0	+ / 0	++ / ++	0 / 0
case 4	i/0	+++ / +++	i/0	+ / 0	+++ / +++	i/0	+ / 0	+++/+++	i/0	+ / +	+++ / ++	i/0
case 5	+ / ++	+++ / +++	0 / 0	+ / 0	+++ / ++	0 / 0	+ / 0	+++ / ++	0 / 0	+ / 0	+++ / ++	0 / 0
case 6	+/+	++ / ++	0 / 0	+ / 0	+++ / ++	0 / 0	+ / 0	++ / ++	0 / 0	+ / 0	+++ / ++	0 / 0
case 7	+/+	++ / ++	0 / 0	+ / 0	++ / +	0 / 0	+ / 0	++ / ++	0 / 0	i / 0	++ / ++	0 / 0
case 8	i/i	+/+	0 / 0	+ / 0	+/+	0 / 0	+ / 0	+/+	0 / 0	+ / 0	+/+	0 / 0
case 9	i/0	++ / ++	0 / 0	i/0	++ / ++	0 / 0	i/0	++ / ++	0 / 0	i/0	+++ / ++	0 / 0
case 10	na	na	0 / 0	+ / 0	+/+	0 / 0	+ / 0	+/+	0 / 0	+ / 0	+/+	0 / 0
case 11	+ / 0	++ / +	0 / 0	+ / 0	+/+	0 / 0	na	na	na	+ / 0	+/+	0 / 0
case 12	+/+	++ / ++	0 / 0	+ / 0	+++ / ++	0 / 0	na	na	na	+ / 0	+++ / ++	0 / 0
case 13	+ / 0	+/+	0 / 0	+ / 0	+/+	0 / 0	+ / 0	+/+	0 / 0	+ / 0	+/+	0 / 0
case 14	i/0	++ / ++	0 / 0	i/0	+++/+++	0 / 0	+ / 0	++ / ++ **	0 / 0	+ / 0	++ / ++ **	0 / 0
case 15	i/0	++ / ++	0 / 0	+ / 0	++ / ++	0 / 0	+ / 0	++ / ++	0 / 0	+ / 0	++ / ++	0 / 0
case 16	i/0	++ / ++	0 / 0	+ / 0	++ / ++	0 / 0	+ / 0	++ / ++	0 / 0	+ / 0	++ / ++	0 / 0
case 17	i/0	++ / ++	0 / 0	i/0	++ / ++	0 / 0	i/0	+++ / +++	0 / 0	i/0	++ / ++	0 / 0
case 18	i/0	++ / ++	0 / 0	+ / 0	+/+	0 / 0	+ / 0	+/++	0 / 0	+ / 0	+/+	0 / 0
case 19	+ / 0	++ / ++	i/0	+ / 0	+++ / ++	0 / 0	+ / 0	+++ / ++	i/0	+ / 0	+++ / ++	i/0
case 20	+ / 0	+ / +	i/0	+ / 0	++ / +	0 / 0	+ / 0	++ / +	0 / 0	+ / 0	++ / +	0 / 0
case 21	+/+	++ / ++	0 / 0	+ / 0	+/+	0 / 0	+ / 0	+/+	0 / 0	+ / 0	+/+	0 / 0
case 22	+ / ++	++ / ++	0 / 0	+ / 0	++/+	+ / 0	+ / 0	+/+	+ / 0	+ / 0	++/+	+ / 0
case 23	i/0	++ / ++	0 / 0	+ / 0	++/+	0 / 0	+ / 0	++ / ++	0 / 0	+ / 0	++/+	+ / 0
case 24	i/0	++ / ++	0 / 0	+ / 0	++ / +	0 / 0	+ / 0	++ / +	0 / 0	+ / 0	++ / +	0 / 0
case 25	+/0	++ / ++	0 / 0	+ / 0	+++ / ++	0 / 0	+ / 0	+++ / ++	0 / 0	+ / 0	+++ / ++	0 / 0
case 26	i/0	+ / +	0 / 0	i/0	+ / +	0 / 0	i/0	+ / +	0 / 0	i/0	++/+	0 / 0
case 27	i/0	++ / ++	0 / 0	i/0	+ / +	0 / 0	i/0	++ / ++	0 / 0	i/0	++ / ++	0 / 0
case 28	+/0	++ / ++	0 / 0	+/0	++ / ++	0 / 0	+/0	++ / ++	0 / 0	+/0	++ / ++	0 / 0
case 29	i/0	++ / ++	0 / 0	i/0	+ / +	0 / 0	i/0	+ / +	0 / 0	+/0	++/+	0 / 0
case 30	+/i	++ / ++	0 / 0	+/++	+++/+++	0 / 0	+/++	+++/+++	0 / 0	i/0	+++ / +++	0 / 0
case 31	i/0	+ / ++	0 / 0	i/0	+ / +	0 / 0	i/0	++ / +	0 / 0	i/0	++ / +	0 / 0
case 32	+/+	++ / ++	0 / 0	++/++	+++/+++	0 / 0	++/++	+++/+++	0 / 0	++/++	+++/+++	0 / 0

	leptomeninges global			white matter supratentorial			frontal			temporal			parietal		
	CD8	HLA-DR	CD20	CD8	HLA-DR	CD20	CD8	HLA-DR	CD20	CD8	HLA-DR	CD20	CD8	HLA-DR	CD20
	perivascular / arachnoidea	perivascular / arachnoidea	perivascular / arachnoidea	perivascular / parenchyma	perivascular / parenchyma	perivascular / parenchyma	perivascular / parenchyma	perivascular / parenchyma	perivascular / parenchyma	perivascular / parenchyma	perivascular / parenchyma	perivascular / parenchyma	perivascular / parenchyma	perivascular / parenchyma	perivascular / parenchyma
case 1	+ / 0	++ / ++	0 / 0	+ / 0	++/+++	0 / 0	+ / 0	++ / ++	0 / 0	+ / 0	+++ / +++	0 / 0	na	na	na
case 2	+ / 0	+ + / + +	i/0	+ / 0	++/+++	0 / 0	++ / 0	+++ / +++	i / 0	+ / 0	+++ / ++	0 / 0	+ / 0	+++ / ++	0 / 0
case 3	+ / 0	+ + / + +	0 / 0	+ / 0	++/+++	0 / 0	+ / 0	++ / ++	0 / 0	+ / 0	++ / ++	0 / 0	+ / 0	++ / ++	0 / 0
case 4	+ / 0	+++ / ++	i/0	+ / 0	++/+++	i/0	+ / 0	+++ / +++	i/0	+ / 0	+++ / +++	i/0	+ / 0	+++ / +++	i/0
case 5	+ / 0	+++ / ++	0 / 0	+ / 0	+++ / ++	0 / 0	+ / 0	+++ / ++	0 / 0	+ / 0	+++ / ++	0 / 0	+ / 0	+++ / ++	0 / 0
case 6	+ /i	+++ / ++	0 / 0	+/- /0	++/+++	0 / 0	+ / 0	+++ / ++	0 / 0	+ / 0	+++ / ++	0 / 0	+ / 0	+++ / ++	0 / 0
case 7	+ / 0	+ + / + +	0 / 0	+ / 0	++/++	0 / 0	+ / 0	++ / ++	0 / 0	+ / 0	++ / ++	0 / 0	+ / 0	++ / ++	0 / 0
case 8	+ / 0	+/+	0 / 0	+ / 0	+/+	0 / 0	+ / 0	+/+	0 / 0	+ / 0	+/+	0 / 0	+ / 0	+/+	0 / 0
case 9	i/0	++ / ++	0 / 0	i/0	++ / ++	0 / 0	i/0	++ / ++	0 / 0	i/0	++ / ++	0 / 0	i/0	++ / ++	0 / 0
case 10	+ / 0	+/+	0 / 0	+ / 0	+/+	0 / 0	+ / 0	+/+	0 / 0	+ / 0	+/+	0 / 0	+ / 0	+/+	0 / 0
case 11	+ / 0	+/+	0 / 0	+ / 0	+/+	0 / 0	+ / 0	++ / +	i / 0	+ / 0	++ / +	0 / 0	+ / 0	++ / +	0 / 0
case 12	+ / 0	+++ / ++	0 / 0	+ / 0	++/+++	0 / 0	+ / 0	+++/+++	i / 0	+ / 0	+++/+++	0 / 0	+ / 0	++ / ++	0 / 0
case 13	+ / 0	+/+	0 / 0	+ / 0	+/+	0 / 0	+ / 0	+/+	0 / 0	+ / 0	+/+	0 / 0	+ / 0	+/+	0 / 0
case 14	i/0	+++/+++	0 / 0	+ / 0	++ / ++	0 / 0	+ / 0	+ + / ++	0 / 0	i/0	+ + / ++	0 / 0	i/0	++ / ++	0 / 0
case 15	+ / 0	+/+	0 / 0	+ / 0	++ / ++	0 / 0	+ / 0	++ / ++	0 / 0	+ / 0	++ / ++	0 / 0	+ / 0	++ / ++	0 / 0
case 16	+ / 0	+/+	0 / 0	+ / 0	++ / ++	0 / 0	+ / 0	++ / +	0 / 0	+ / 0	++ / ++	0 / 0	+ / 0	++ / ++	0 / 0
case 17	i/0	++ / ++	0 / 0	i/0	++ / ++	0 / 0	i/0	++ / ++	0 / 0	i/0	++ / ++	0 / 0	i/0	++ / ++	0 / 0
case 18	+ / 0	+/+	0 / 0	+ / 0	++/++	0 / 0	+ / 0	+/+	0 / 0	+ / 0	+/+	0 / 0	+ / 0	+/+	0 / 0
case 19	+ / 0	+/+	0 / 0	+ / 0	++ / ++	0 / 0	+ / 0	+/+	0 / 0	+ / 0	++ / ++	i/0	+ / 0	++ / ++	0 / 0
case 20	+ / 0	++/+	0 / 0	+ / 0	++ / +	i/0	+ / 0	+ / +	i/0	+ / 0	++ / +	0 / 0	+ / 0	+++ / +++	0 / 0
case 21	+ / 0	+/+	0 / 0	+ / 0	++ / ++	0 / 0	+ / 0	+ / +	0 / 0	+ / 0	+ / +	0 / 0	+ / 0	+ / +	0 / 0
case 22	+ / 0	++/+	+ / 0	+ / 0	++/+	0 / 0	+ / 0	+ / +	0 / 0	+ / 0	++/+	+ / 0	++ / 0	++ / ++	+ / 0
case 23	+ / 0	++/+	0 / 0	+ / 0	++ / ++	0 / 0	+ / 0	++ / ++	0 / 0	+ / 0	++ / ++	0 / 0	+ / 0	++ / ++	0 / 0
case 24	+ / 0	++ / +	0 / 0	+ / 0	++ / ++	0 / 0	+ / 0	++ /+ +	0 / 0	+ / 0	++ / +	0 / 0	+ / 0	++ / ++	0 / 0
case 25	+ / 0	+++ / ++	0 / 0	+ / 0	+++ / ++	0 / 0	+ / 0	+++ / +++	0 / 0	+ / 0	+++ / +++	0 / 0	+ / 0	+++ / +++	0 / 0
case 26	i/0	+ / +	0 / 0	+ / 0	++/+	0 / 0	i/0	+ / +	0 / 0	i/0	+ / +	0 / 0	i/0	+ / +	0 / 0
case 27	i/0	+ / +	0 / 0	+ / 0	++/+	0 / 0	i/0	+ / +	0 / 0	i/0	+ / +	0 / 0	i/0	++ / ++	0 / 0
case 28	+/0	++ / ++	0 / 0	+ / 0	++ / +	0 / 0	+ / 0	++ / +	0 / 0	+/0	++ / ++	0 / 0	+/0	++ / ++	0 / 0
case 29	i/0	+ / +	0 / 0	i / 0	++ / ++	0 / 0	i / 0	++ /+ +	0 / 0	i / 0	++ /+ +	0 / 0	i / 0	++ /+ +	0 / 0
case 30	i/0	+++ / +++	0 / 0	i/0	+++ / +++	0 / 0	i/0	+++ / +++	0 / 0	i/0	+++ / +++	0 / 0	i/0	+++ / +++	0 / 0
case 31	i/0	+ / +	0 / 0	i/0	+ / +	0 / 0	i/0	+ / +	0 / 0	i/0	+ / +	0 / 0	i/0	++ / +	0 / 0
case 32	+/+	++ / ++	0 / 0	+ / 0	+++ / +++	0 / 0	++ / +	++ /+ +	0 / 0	++ /+ +	+++ / +++	0 / 0	i / 0	++ /+ +	0 / 0

	occipital			hypothalamus			basal ganglia			thalamus			midbrain / s nigra		
	CD8	HLA-DR	CD20	CD8	HLA-DR	CD20	CD8	HLA-DR	CD20	CD8	HLA-DR	CD20	CD8	HLA-DR	CD20
	perivascular / parenchyma	perivascular / parenchyma	perivascular / parenchyma	perivascular / parenchyma	perivascular / parenchyma	perivascular / parenchyma	perivascular / parenchyma	perivascular / parenchyma	perivascular / parenchyma	perivascular / parenchyma	perivascular / parenchyma	perivascular / parenchyma	perivascular / parenchyma	perivascular / parenchyma	perivascular / parenchyma
case 1	na	na	na	na	na	na	+ / 0	++ / ++	0 / 0	na	na	na	na	na	na
case 2	+ / 0	+++ / ++	0 / 0	++ / i	+ ++ / ++	0 / 0	+ / 0	++ / ++	0 / 0	+ / +	+++/++	i/0	+ / 0	++/+++	i/0
case 3	+ / 0	++ / ++	0 / 0	+ / 0	++ / +	0 / 0	+ / 0	++ / +	0 / 0	+ / 0	++ / +	0 / 0	i / 0	++ / +	0 / 0
case 4	+ / 0	+++ / +++	i/0	+ / 0	+++ / +++	i/0	+ / 0	+++ / +++	i/0	+ / 0	+++ / +++	i/0	+ / 0	+++ / +++	i/0
case 5	+ / 0	+++ / ++	0 / 0	+ / 0	+++ / ++	0 / 0	+ / 0	+++ / ++	0 / 0	+ / 0	+++ / ++	0 / 0	+ / +	+++ / +++	0 / 0
case 6	+ / 0	+++ / ++	0 / 0	+ / 0	+++ / ++	0 / 0	+ / 0	++ / ++	0 / 0	+ / 0	+++ / ++	0 / 0	i / 0	++/++	0 / 0
case 7	+ / 0	++ / ++	0 / 0	+ / 0	++/++	0 / 0	+ / 0	+ / +	0 / 0	+ / 0	+ / +	0 / 0	+ / +	++/++	0 / 0
case 8	+ / 0	+/+	0 / 0	+ / 0	+/+	0 / 0	+ / 0	+/+	0 / 0	+ / 0	+/+	0 / 0	+ / 0	+/+	0 / 0
case 9	i/0	++ / ++	0 / 0	i/0	++ / ++	0 / 0	i/0	++ / ++	0 / 0	i/0	++ / ++	0 / 0	i/0	+++ / +++	0 / 0
case 10	+ / 0	+/+	0 / 0	+ / 0	+/+	0 / 0	+ / 0	+/+	0 / 0	+ / 0	+/+	0 / 0	+ / 0	+/+	0 / 0
case 11	+ / 0	+/+	i / 0	+ / 0	+/+	0 / 0	+ / 0	+/+	0/0	+ / 0	+/+	0 / 0	+ / 0	+/+	0 / 0
case 12	+ / +	+++/+++	0 / 0	+ / 0	++ / ++	0 / 0	+ / 0	++ / ++	0/0	+ / 0	+++/++	0 / 0	+ / 0	++/++	i / 0
case 13	+ / 0	+/+	0 / 0	+ / 0	+/+	0 / 0	+ / 0	+/+	0 / 0	+ / 0	+/+	0 / 0	+ / 0	+/+	v
case 14	i/0	++ / ++	0 / 0	i/0	++ / ++	0 / 0	+ / 0	++ / ++	0/0	i/0	++ / ++	0 / 0	+ / 0	++/++	0 / 0
case 15	+ / 0	++ / ++	0 / 0	+ / 0	++ / ++	0 / 0	+ / 0	++ / ++	0 / 0	+ / 0	++ / ++	0 / 0	+ / 0	++ / ++	0 / 0
case 16	+ / 0	++ / ++	0 / 0	+ / 0	+++ / +++	0 / 0	+ / 0	++ / ++	0 / 0	+ / 0	++ / ++	0 / 0	+ / 0	++ / +++	0 / 0
case 17	i/0	++ / ++	0 / 0	i/0	++ / ++	0 / 0	i/0	++ / ++	0 / 0	i/0	++ / ++	0 / 0	i/0	++ / ++	0 / 0
case 18	+ / 0	+/+	0 / 0	+ / 0	+/+	0 / 0	+ / 0	+/+	i/0	+ / 0	+/+	i/0	i/0	++ / ++	0 / 0
case 19	+ / 0	++ / ++	0 / 0	+ / 0	++ / ++	0 / 0	+ / 0	++ / ++	0 / 0	+ / 0	++ / ++	i/0	+ / 0	++ / ++	0 / 0
case 20	+ / 0	++ / +	0 / 0	+ / 0	++ / ++	0 / 0	+ / 0	+ / +	i/0	+ / 0	++ / +	0 / 0	+ / 0	++ /+ +	0 / 0
case 21	+ / 0	+ / +	0 / 0	+ / 0	+ / +	0 / 0	+ / 0	+ / +	0 / 0	+ / 0	+ / +	0 / 0	+/+	++ / ++	0 / 0
case 22	+ / 0	+++ / +++	0 / 0	+ / 0	+++ / +++	0 / 0	+ / 0	++ / ++	+ / +	+ / 0	++/+	0 / 0	+/+	++ / ++	0 /i
case 23	+ / 0	++ / ++ wm	0 / 0	+ / 0 *	++ / ++ *	0 / 0	+ / 0	++ / ++	0 / 0	+ / 0	++ / ++	0 / 0	+ / 0	++ / ++	0 / 0
case 24	+ / 0	++ / +	0 / 0	+ / 0	+++ / +++	0 / 0	+ / 0	++ /+ +	0 / 0	+ / 0	++ /+ +	0 / 0	+ / 0	++ / ++	0 / 0
case 25	+ / 0	++ / ++	0 / 0	+ / +	+++ / +++	0 / 0	+ / 0	+++/+++	0 / 0	+ / 0	+++/+++	0 / 0	+ / 0	+++/+++	0 / 0
case 26	i/0	+ / +	0 / 0	i/0	+ / +	0 / 0	+ / 0	++ / ++	0 / 0	i/0	+ / +	0 / 0	+ / 0	++ / ++	0 / 0
case 27	i/0	++ / ++	0 / 0	i/0	++ / ++	0 / 0	i/0	++ / ++	0 / 0	i/0	+ / +	0 / 0	+ / 0	++ / ++	0 / 0
case 28	+/0	++ / ++	0 / 0	+/0	++ / ++	0 / 0	+/0	++ / ++	0 / 0	+/0	++ / ++	0 / 0	i / 0	++ / ++	0 / 0
case 29	i / 0	++ /+ +	0 / 0	i / 0	++ /+ +	0 / 0	i / 0	++ /+ +	0 / 0	+ / 0	++ /+ +	0 / 0	i/0	+ / +	0 / 0
case 30	i/0	+++ / +++	0 / 0	i/0	+++ / +++	0 / 0	i / 0	+++/+++	0 / 0	i/0	+++ / +++	0 / 0	i/0	+++ / +++	0 / 0
case 31	i/0	++ / +	0 / 0	i/0	+ / +	0 / 0	i/0	+ / +	0 / 0	i/0	+ / +	0 / 0	i/0	++ / +	0 / 0
case 32	i / 0	++ /+ +	0 / 0	++ /+ +	+++/+++	0 / 0	++ /+	+++/+++	0 / 0	++ /+ +	+++/+++	0 / 0	+/+	+++ / +++	0 / 0

	pons			medulla oblongata			cerebellum cortex/white matter			cerebellar dentate		
	CD8	HLA-DR	CD20	CD8	HLA-DR	CD20	CD8	HLA-DR	CD20	CD8	HLA-DR	CD20
	perivascular / parenchyma	perivascular / parenchyma	perivascular / parenchyma	perivascular / parenchyma	perivascular / parenchyma	perivascular / parenchyma	perivascular / parenchyma	perivascular / parenchyma	perivascular / parenchyma	perivascular / parenchyma	perivascular / parenchyma	perivascular / parenchyma
case 1	na	na	na	na	na	na	na	na	na	na	na	na
case 2	+ / +	++/++	i / 0	++ / + *	+++ / +++ *	i / 0	++ / i	+++ / ++	0 / 0	++ / +	+++ / ++	0 / 0
case 3	+ / 0	++ / +	0 / 0	++ / ++ *	++ / ++ *	0 / 0	++ / +	++ / ++	0 / 0	++ / +	+++ / ++	0 / 0
case 4	+ / 0	+++/+++	i/0	+ / ++ *	+++ / +++ *	0 / 0	+ / 0	+++ / +++	0 / 0	+ / 0	+++ / +++	0 / 0
case 5	+ / + *	+++ / +++	0 / 0	+ / ++ **	+++ / +++ *	+ / 0	+ / 0	+++ / ++	0 / 0	+ / 0	+++ / ++	0 / 0
case 6	++ /+ +	+++ / ++	0 / 0	++ /+ +	+++ / ++	0 / 0	+ / 0	+++ / ++	0 / 0	+ / 0	+++ / ++	0 / 0
case 7	+ / +	++/++	0 / 0	++ / ++ *	+++ / ++	i / 0	+ / 0	++/++	0 / 0	+ / 0	+++ / ++	0 / 0
case 8	+ / 0	+/+	0 / 0	+ / 0	+/+	0 / 0	+ / 0	+/+	0 / 0	+ / 0	+/+	0 / 0
case 9	i/0	+++ / +++	0 / 0	i/0	+++ / +++	0 / 0	i/0	++ / ++	0 / 0	i/0	+++ / +++	0 / 0
case 10	+ / 0	+/+	0 / 0	+ / 0	+/+	0 / 0	+ / 0	+/+	0 / 0	+ / 0	+/+	0 / 0
case 11	+ / 0	+/+	0 / 0	+ / 0	++ / +	0 / 0	+ / 0	++ / +	0 / 0	+ / 0	++ / +	0 / 0
case 12	+ / 0	++/++	0 / 0	+ / +	++/++	0 / 0	+ / 0	+++ / +++	0 / 0	+ / 0	+++ / +++	0 / 0
case 13	+ / 0	+/+	0 / 0	+ / 0	+/+	0 / 0	+ / 0	+/+	0 / 0	+ / 0	+/+	0 / 0
case 14	+ / 0	++/++	0 / 0	+ / 0	+++ / +++	0 / 0	+ / 0	++ / ++	0 / 0	i / 0	++ / ++	0 / 0
case 15	+ / 0	++ / ++	0 / 0	+ / 0	++ / ++	0 / 0	+ / 0	++ / ++	0 / 0	+ / 0	++ / ++	0 / 0
case 16	+ / 0	++ / +++	0 / 0	+ / 0	++ / +++	0 / 0	+ / 0	++ / ++	0 / 0	+ / 0	++ / ++	0 / 0
case 17	i/0	++ / ++	0 / 0	i/0	++ / ++	0 / 0	i/0	++ / ++	0 / 0	+ / 0	++ / ++	0 / 0
case 18	+ / 0	++ / ++	0 / 0	+ / 0	++ / ++	0 / 0	+ / 0	++ / ++	0 / 0	+ / 0	++ / ++	0 / 0
case 19	+ / 0	+++ / +++	0 / 0	++ / +	+++ / +++	0 / 0	+ / 0	+++ / ++	0 / 0	+ / 0	+++ / ++	0 / 0
case 20	+ / 0	++ /+ +	0 / 0	+ / 0	++ /+ +	0 / 0	+ / 0	++ / +	0 / 0	+ / 0	++ / ++	i / 0
case 21	+/+	++ / ++	0 / 0	++/++	++ / ++	0 / 0	+ / 0	+ / +	0 / 0	+ / 0	++ / ++	0 / 0
case 22	+/i	++ / ++	i /0	+/i	++ / +	i /0	++/0	++ / +	i /0	+/i	++ / + (small nodule wm)	i /0
case 23	+ / 0	++ / ++	0 / 0	+ / 0	++ / ++	0 / 0	+ / 0	++ / ++	0 / 0	+ / 0	++ / ++	0 / 0
case 24	+ / 0	+++/+++	0 / 0	+ / 0	++ / ++	0 / 0	+ / 0	++ / ++	0 / 0	+ / 0	++ / ++	0 / 0
case 25	+ / 0	+++/+++	0 / 0	+ / 0	+++/+++	0 / 0	+ / 0	+++/+++	0 / 0	+ / 0	+++/+++	0 / 0
case 26	+ / 0	++ / ++	0 / 0	+ / 0	++ / ++ *	i /0	+ / 0	++ / ++	0 / 0	+ / 0	++ / ++	0 / 0
case 27	+ / 0	+++ / ++	0 / 0	+ / 0	++ / ++	0 / 0	+ / 0	++ / ++	0 / 0	+ / 0	++ / + (small nodules)	0 / 0
case 28	i / 0	++ / ++	0 / 0	i / 0	++ / ++	0 / 0	i / 0	++ / ++	0 / 0	i / 0	++ / ++	0 / 0
case 29	i / 0	++ /+ +	0 / 0	i / 0	++ /+ +	0 / 0	i / 0	++ /+ +	0 / 0	i / 0	++ /+ +	0 / 0
case 30	+/+	+++ / +++	0 / 0	+/+	+++ / +++	0 / 0	+/+	+++ / +++	0 / 0	+/+	+++ / +++	0 / 0
case 31	i/0	++ / +	0 / 0	i/0	++ / ++	0 / 0	i/0	++ / +	0 / 0	i/0	++ / +	0 / 0
case 32	+/+	+++ / +++	0 / 0	+/+	+++ / +++	0 / 0	+ / 0	++ / ++	0 / 0	+ / 0	++ / ++	0 / 0

* nodules
** neuritis
